# IL-1β Pathway Inhibition in Asthma and COPD: Strong Biological Rationale, Disappointing Clinical Trials, and Emerging New Opportunities

**DOI:** 10.1007/s00408-026-00897-9

**Published:** 2026-05-22

**Authors:** Mario Cazzola, Clive P. Page, Maria Gabriella Matera

**Affiliations:** 1 Unit of Respiratory Medicine, Department of Experimental Medicine, University of Rome ‘Tor Vergata’, Rome, Italy; 2 Institute of Pharmaceutical Science, King’s College London, London, UK; 3 Unit of Pharmacology, Department of Experimental Medicine, University of Campania ‘Luigi Vanvitelli’, Naples, Italy

**Keywords:** Asthma and COPD endotypes, Interleukin-1β, Neutrophilic inflammation, NLRP3 inflammasome, Precision medicine

## Abstract

Interleukin-1β (IL-1β) is a key mediator of innate immunity and a central driver of airway inflammation in asthma and chronic obstructive pulmonary disease (COPD). Elevated IL-1β levels in sputum, bronchoalveolar lavage, and airway tissues correlate with neutrophilic inflammation, exacerbation frequency, airflow limitation, and steroid resistance. Mechanistically, IL-1β promotes epithelial activation, epithelial–mesenchymal transition, neutrophil recruitment, inflammasome activation, and immune cell plasticity, particularly driving ILC2 transdifferentiation toward pro-inflammatory phenotypes. Despite strong biological rationale, clinical trials targeting IL-1 signaling through receptor blockade or IL-1β neutralization have yielded limited benefits in certain patient populations. Therapeutic failure is largely attributed to disease heterogeneity, lack of biomarker-guided stratification, redundant inflammatory pathways, and suboptimal timing of intervention. Emerging strategies include precision medicine approaches with biomarker enrichment, upstream NLRP3 inflammasome inhibition, combinatorial cytokine targeting, modulation of signaling intermediates, temporally targeted therapy during exacerbations, and localized airway delivery systems. Integration of multi-omics profiling and endotype-based patient selection may enhance therapeutic responsiveness. Future clinical trials should adopt adaptive designs to validate IL-1-targeted interventions in biologically defined subgroups of patients with asthma or COPD.

## The Biological Importance of IL-1β in Asthma and COPD

Interleukin-1 beta (IL-1β) is a key mediator of innate immunity and one of the most potent pro-inflammatory cytokines involved in host defense and tissue injury. Although IL-1β plays an essential role in protecting against pathogens [1], it has also emerged as a central driver of chronic inflammation when dysregulated. In chronic airway diseases, such as asthma and chronic obstructive pulmonary disease (COPD), IL-1β is increasingly recognized as an upstream regulator of neutrophilic inflammation, epithelial activation, and structural remodeling of the airways [2]. The evidence supporting these roles derives from complementary methodological sources, including human airway studies, preclinical animal models, and in vitro or ex vivo experimental systems, which are explicitly contextualized to improve translational interpretation.

Clinical studies have repeatedly shown that IL-1β levels are elevated in induced sputum, bronchoalveolar lavage fluid, and bronchial biopsy specimens from patients with asthma or COPD [3, 4]. However, the proportion of patients with asthma or COPD in whom IL-1β is increased in the airways appears to be relatively small and difficult to identify using non-airway measures. Large-scale multicenter studies from networks such as SARP (Severe Asthma Research Program) and UBIOPRED (Unbiased Biomarkers for the Prediction of Respiratory Disease Outcomes) [5, 6] have not emphasized IL-1β dysregulation as a prominent finding across the broader asthma population. Increased airway IL-1β expression correlates with higher neutrophil counts, more severe airflow limitation, and a greater risk of exacerbations [4, 7]. In SARP data, extracellular DNA (eDNA) traps and IL-1β occur in the same subsets of patients with severe asthma, representing approximately 13% of the cohort who are “eDNA-high” [8]. In asthma, this association is especially pronounced in neutrophilic or type (T)2-low phenotypes, where IL-1β levels parallel sputum neutrophilia and reduced responsiveness to corticosteroid therapy [9]. Histological analyses further reveal enhanced IL-1β expression within bronchial mucosa compared with healthy controls, supporting its involvement in sustained airway inflammation [3]. In COPD, similar patterns are observed. IL-1β supports ongoing neutrophilic airway inflammation, mucus hypersecretion, and possibly emphysematous remodeling of lung tissue. IL-1β concentrations rise further during acute exacerbations and are frequently associated with increased bacterial load and airway neutrophilia [7]. During acute exacerbations, it remains unclear whether elevated IL-1β is driving the exacerbation or simply represents a normal host defense response against viruses or bacteria [2]. Beyond serving as an inflammatory marker, IL-1β appears to actively contribute to disease progression, with airway and systemic IL-1β activity linked to exacerbation frequency and overall disease severity [4].

**Fig. 1:**
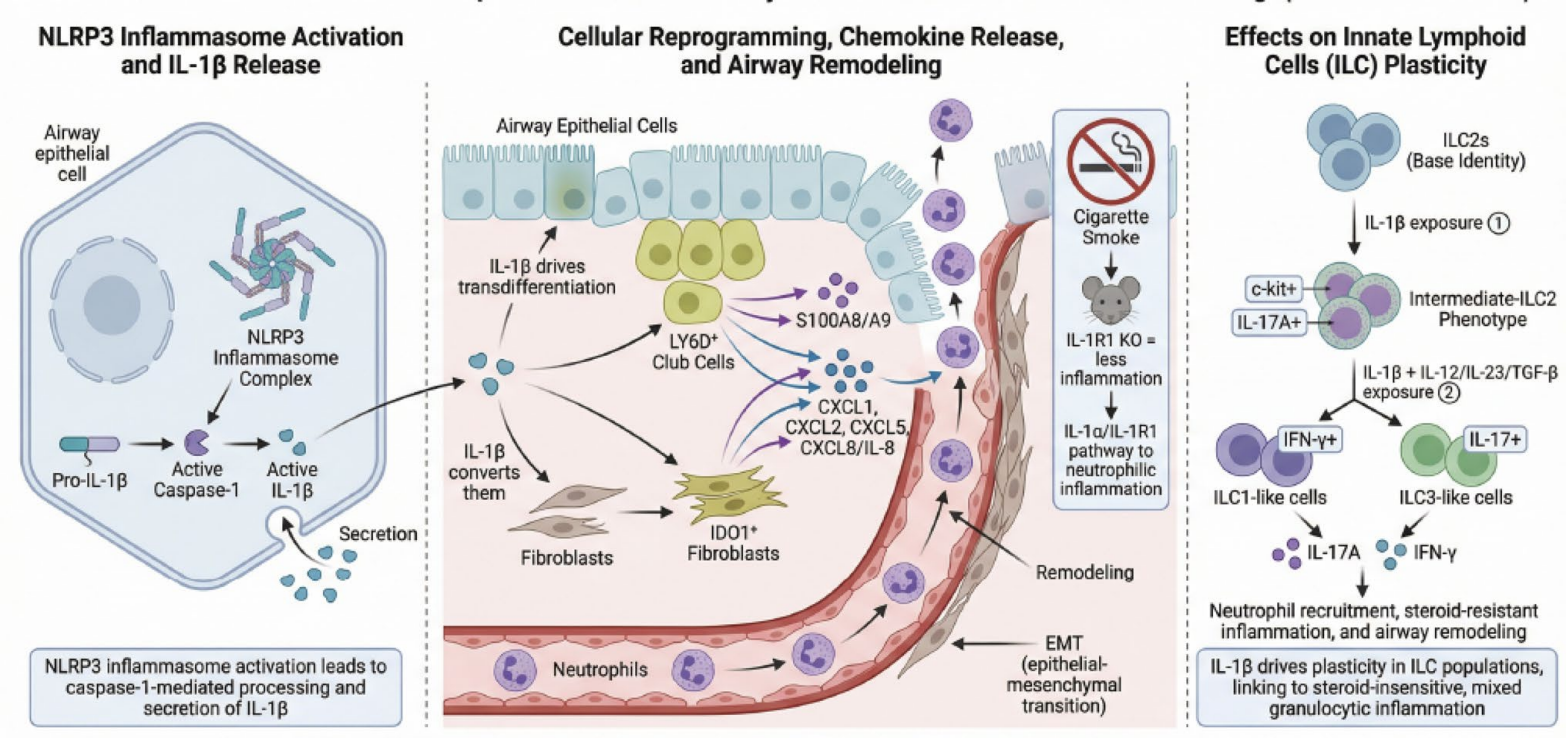
Mechanistic role of IL-1β in chronic airway inflammation and remodeling in asthma and COPD. IL-1β activates NLRP3-dependent inflammatory signaling pathways, drives cellular reprogramming, and promotes the release of proinflammatory cytokines and chemokines, as well as ILC plasticity. These mechanisms converge to sustain neutrophilic inflammation, airway remodeling, and steroid-resistant disease

Mechanistically, IL-1β is known to drive neutrophilic inflammation, promote epithelial-mesenchymal transition, and induce the release of C-X-C motif chemokine ligands (CXCLs), such as CXCL1, CXCL2, CXCL5, and CXCL8 (also known as IL-8) from fibroblasts, contributing to airway remodeling and neutrophil recruitment [2, 10] (Fig. 1**)**. Neutrophilic inflammation in the airways involves multiple cytokines and chemokines beyond IL-1β, including tumor necrosis factor (TNF)-α, IL-6, IL-17 A, granulocyte macrophage-colony stimulating factor, and granulocyte-colony stimulating factor (G-CSF), which collectively regulate neutrophil recruitment and activation [11, 12].

These mechanistic insights are derived from a combination of human airway data and experimental systems. In this context, epithelial–stromal signaling should be viewed as a central bidirectional regulatory axis, in which epithelial cells and fibroblasts engage in continuous crosstalk that amplifies inflammatory signaling and drives airway remodeling, rather than functioning as independent effector compartments. IL-1β acts as a key upstream mediator of this epithelial–stromal loop, reinforcing both epithelial activation and fibroblast-derived chemokine production.

Recent studies in chronic airway disease models have demonstrated that IL-1β promotes the transdifferentiation of airway epithelial cells into LY6D-positive club cells and fibroblasts into indoleamine 2,3-dioxygenase 1-positive fibroblasts. These reprogrammed cell populations enhance neutrophil recruitment through increased production of S100A8/A9 and CXCLs [13]. These findings are largely derived from preclinical airway models and in vitro/ex vivo systems, primarily including nasal airway experimental models, and their translational relevance to distal human bronchial and alveolar compartments remains inferential.

IL-1β promotes plasticity in innate lymphoid cells (ILCs) by driving the transdifferentiation of ILC2s toward intermediate or alternative phenotypes. Specifically, IL-1β induces an intermediate-ILC2 population that co-expresses T2 and T3 markers (e.g., c-kit and IL-17 A), which is associated with mixed granulocytic inflammation and airway neutrophilia, providing a mechanistic link to steroid-resistant, neutrophilic asthma endotypes [14]. In the presence of additional cytokines, such as IL-12, IL-23, or transforming growth factor-beta (TGF-β), IL-1β further directs the conversion of ILC2s toward ILC1- or ILC3-like phenotypes, a shift that enhances production of interferon-γ (IFN-γ) and IL-17 [15]. The biology of IL-1β in the airway should be considered in the context of the broader T1 immune response. SARP data demonstrate that the T1 immune pathway, which includes IL-1β but is dominated by IFN-γ, is upregulated in some asthma patients. This pathway is associated with increased asthma severity, corticosteroid resistance, and airway hyperreactivity [16]. This functional reprogramming amplifies steroid-resistant neutrophilic inflammation and contributes to airway remodeling [17, 18]. It is important to note that neutrophilia in the airways is not orthogonal to eosinophilia [19]. While some T2-low patients exhibit neutrophilic inflammation, many do not. Similarly, some T2-high patients have airway neutrophilia while others do not, indicating that these inflammatory patterns can coexist and overlap [20]. Preclinical evidence shows that IL-1α/IL-1 receptor 1 (IL-1R1) signaling drives cigarette smoke-induced neutrophilic inflammation and that IL-1R1 knockout mice demonstrate reduced smoke-induced airway inflammation [21].

The maturation and secretion of IL-1β depend on activation of the pyrin domain-containing 3 (NLRP3) inflammasome, which leads to caspase-1–mediated cleavage of pro-IL-1β into its active form, resulting in increased IL-1β release. This process amplifies airway inflammation, promotes neutrophil recruitment, and contributes to disease severity and steroid resistance in patients with severe asthma or COPD [22, 23].

## Translation into Clinical Trials: A Logical but Unsuccessful Step

The robust pathophysiological evidence linking IL-1β to airway inflammation naturally led to clinical studies of drugs targeting this pathway in patients with asthma or COPD.

### IL-1 Receptor Blockade

The monoclonal antibody (mAb) MEDI8968, targeting IL-1R1, was evaluated in a Phase 2 randomized, double-blind, placebo-controlled trial in 324 patients with COPD at high risk of exacerbations (≥ 2 exacerbations in the past year) receiving standard maintenance therapy [24]. However, the study failed to demonstrate a significant reduction in exacerbation rates (0.71 vs. 0.78 events/year), a clinically meaningful improvement in lung function, or a clear benefit in patient-reported outcomes.

Anakinra, a recombinant IL-1R1 antagonist, demonstrated a significant reduction of endotoxin-induced airway neutrophilia in healthy volunteers [25]. Nevertheless, no adequately powered randomized trials in patients with obstructive lung diseases have been completed, and robust efficacy data in asthma or COPD populations remain lacking.

### Direct IL-1β Neutralization

Canakinumab, a fully human mAb that neutralizes IL-1β, has been studied in small clinical trials that have yielded disappointing or inconclusive results [26]. In a single randomized, double-blind trial involving 16 patients with mild asthma, participants received two administrations of this mAb on days 1 and 15 while continuing their usual treatments. Allergen challenge was performed on days 0 and 28. Canakinumab reduced the late asthmatic response by 28% and significantly lowered circulating IL-1β levels during the observation period, suggesting biological activity and target engagement despite the small sample size [27]. In a larger phase 1/2 study including 147 participants with COPD, the effects of intravenous canakinumab administered at weeks 1, 5, 7, and every 4 weeks thereafter for 45 weeks were assessed [26]. In this clinical trial, canakinumab did not produce a significant improvement in lung function compared with placebo, indicating no efficacy in this patient population.

## Why did These Studies Fail?

**Fig. 2:**
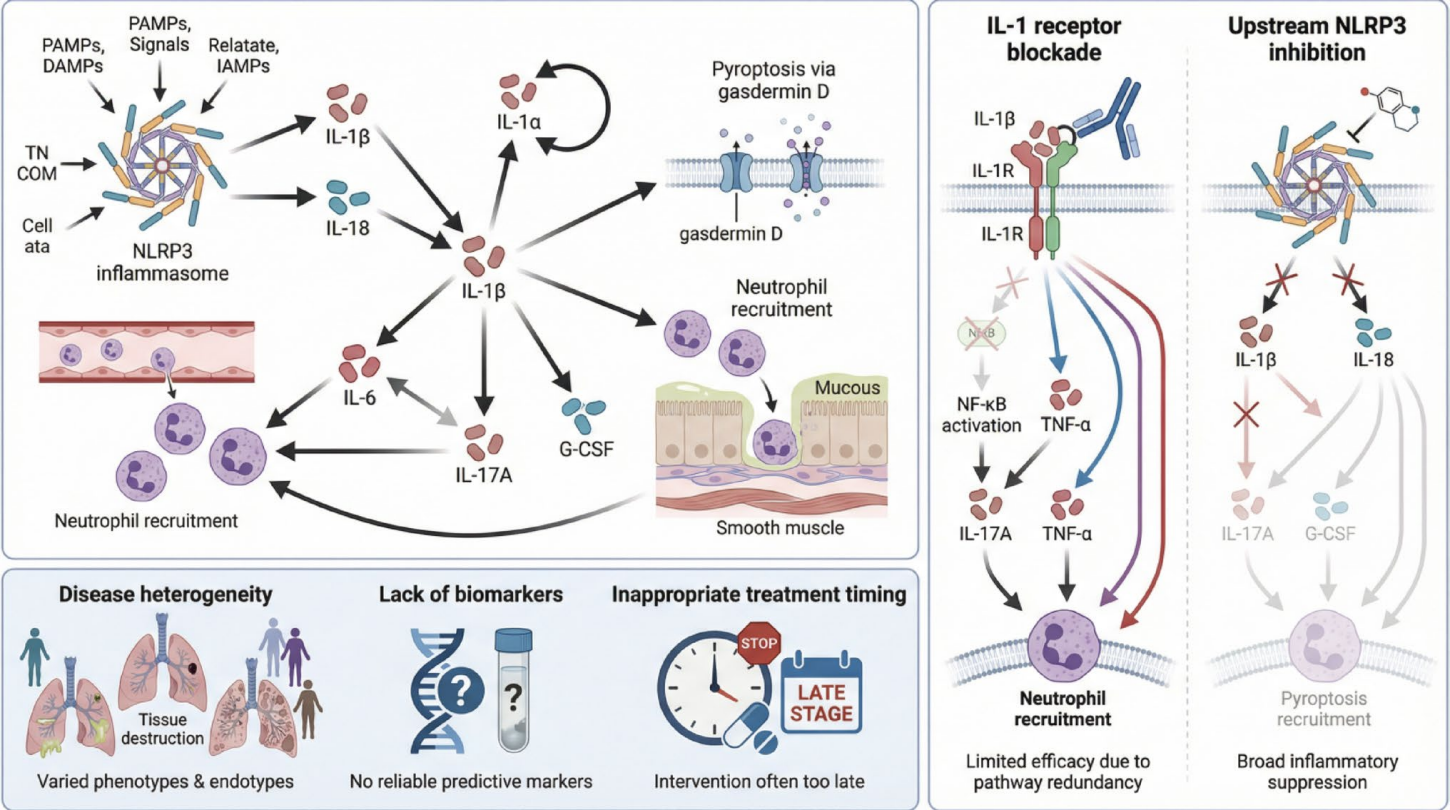
Mechanisms underlying the limited efficacy of IL-1–targeted therapies in asthma and COPD. The figure illustrates the highly interconnected IL-1β–driven inflammatory network and its compensatory pathways, alongside key limitations of clinical trials, including insufficient patient stratification and lack of validated biomarker guidance. It further contrasts downstream IL-1 receptor blockade with upstream targeting of NLRP3 inflammasome activation as alternative therapeutic strategies

Several interconnected explanations may account for the negative clinical outcomes of IL-1 pathway inhibition in patients with COPD or asthma, ranging from inadequate patient stratification to fundamental gaps in understanding relevant disease biology (Fig. 2).

### Disease Heterogeneity and Absence of Validated Biomarkers

Asthma and COPD are highly heterogeneous conditions with distinct inflammatory endotypes. IL-1β may be critical only in specific subgroups (e.g., neutrophilic/T2-low phenotypes), yet most trials so far have enrolled broad COPD or asthma populations without precise molecular stratification [2]. Unfortunately, no validated accessible biomarker exists to identify IL-1-driven disease in clinical practice [28].

### Redundant Inflammatory Pathways and Compensatory Mechanisms

A major limitation of IL-1β-targeted therapies in patients with asthma or COPD likely lies in the intrinsic redundancy of the inflammatory network. IL-1β functions not as an isolated upstream trigger, but as part of a tightly interconnected cytokine system. Blocking IL-1 signaling alone may therefore be insufficient to suppress neutrophilic inflammation when parallel pathways remain active [2]. The IL-1 family itself includes multiple ligands, such as IL-1α and IL-18, as well as signaling and decoy receptors, allowing partial compensation when IL-1β is inhibited [29]. In addition, compensatory upregulation of IL-17 A and G-CSF may sustain neutrophil recruitment despite IL-1 blockade [30]. Importantly, IL-1β operates alongside IL-6 and TNF-α within a core inflammatory cascade that amplifies innate immunity and drives acute-phase responses [31]. In patients with COPD, persistent elevation of IL-6, especially in frequent exacerbators, suggests that targeting IL-1 alone may leave other dominant inflammatory pathways intact [4].

Taken together, the limited clinical efficacy observed with IL-1 inhibitors may reflect not a flawed biological rationale, but the complexity and redundancy characteristic of the cytokine network in chronic airway disease.

### Timing and Disease Stage: Exacerbations Versus Stable Disease

IL-1β may play a greater role in acute inflammatory amplification or during exacerbations rather than in stable chronic disease [2, 32]. The IL-1β-systemic inflammatory axis mediates a vicious cycle in which prior exacerbations elevate baseline IL-1β levels, which in turn predict future exacerbations in COPD [4]. This temporal pattern suggests that IL-1 inhibition might be most effective as a treatment of acute exacerbations rather than as chronic maintenance therapy. However, the pro-inflammatory exacerbation endotype characterized by high IL-1β and TNF-α levels represents a specific target population that has never been prospectively enrolled in IL-1 inhibitor trials [33].

### Upstream Versus Downstream Targeting: The Inflammasome Hypothesis

Targeting IL-1R1 or IL-1β itself may be less effective than inhibiting upstream inflammasome activation, which controls IL-1β maturation, release, and broader inflammatory mechanisms [22]. The NLRP3 inflammasome regulates caspase-1-dependent maturation of IL-1β and IL-18 and induces gasdermin D–mediated pyroptosis, amplifying inflammation beyond the actions of IL-1β alone [34, 35]. In preclinical models of cigarette smoke exposure and steroid-resistant asthma, the NLRP3 inhibitor MCC950 reduced neutrophilic inflammation, mucus production, and IL-1β release more effectively than IL-1β blockade [22]. NLRP3 deficiency similarly attenuated smoke-induced lung inflammation [34]. Human data support these findings, as enhanced NLRP3 activation and IL-1β production correlate with neutrophilic inflammation, disease severity, and steroid resistance in asthma [36].

## Emerging Therapeutic Strategies to Overcome IL-1 Pathway Limitations

Although first-generation IL-1-targeted approaches have yielded disappointing clinical results, advances in understanding disease endotypes, inflammasome biology, and immune plasticity have opened new therapeutic avenues that may enable more effective therapeutic exploitation of this pathway in asthma and COPD (Table 1). These approaches span different levels of translational evidence, ranging from clinically validated therapies in non-airway diseases to preclinical and conceptual strategies not yet tested in asthma or COPD.

**Table 1:** Emerging therapeutic strategies targeting the IL-1 pathway in asthma and COPD

Strategy	Mechanistic rationale	Key biomarkers/targets	Current evidence status	Main limitations/risks
Precision medicine and biomarker stratification	Enrich for IL-1–driven endotypes to improve therapeutic signal	Sputum IL-1β, NLRP3/caspase-1 signatures, neutrophilia, CRP, IL-6, FeNO, blood eosinophils	Supported by transcriptomic/proteomic profiling and exploratory trials	Heterogeneity of biomarkers; lack of validated thresholds
Upstream inflammasome inhibition	Block NLRP3 activation to prevent IL-1β/IL-18 maturation and pyroptosis	NLRP3, inflammasome components	Strong preclinical data; early-phase clinical development (next-gen inhibitors)	Safety concerns, hepatotoxicity (first-gen inhibitors), incomplete clinical validation
Dipeptidyl peptidase 1 inhibition	Inhibition of neutrophil serine protease activation during granulocyte maturation, reducing protease-driven IL-1β activation independently of inflammasome signaling	DPP-1 (cathepsin C), neutrophil elastase, proteinase 3, cathepsin G	Phase 3 evidence in bronchiectasis; FDA-approved oral inhibitor brensocatib (ASPEN trial); translational relevance to neutrophilic airway disease	Limited direct evidence in asthma/COPD; long-term safety and infection risk; indirect IL-1 modulation
Dual/combinatorial cytokine targeting	Counteract inflammatory redundancy via multi-cytokine blockade	IL-1β + IL-17 A / TNF-α / IL-6 / G-CSF	Preclinical support; biologic combinations conceptually feasible	Infection risk; limited clinical data in airway disease
Targeting signaling intermediates (MyD88/IRAK4/NF-κB)	Modulate downstream amplification while preserving partial innate immunity	MyD88, IRAK4, NF-κB pathway	Early-stage small-molecule inhibitors under investigation	Risk of impaired host defense; clinical efficacy unproven
Modulating innate immune cell plasticity	Prevent IL-1β-driven ILC and epithelial/fibroblast transdifferentiation	ILC phenotype stability, metabolic/epigenetic regulators	Experimental/preclinical evidence	Mechanistic complexity; no clinical trials yet
Temporal (exacerbation-focused) targeting	Short-term IL-1 blockade during IL-1-high exacerbations to suppress inflammatory amplification	Exacerbation IL-1β/TNF-α signature; bacteria-associated endotype	Supported by biomarker-defined exacerbation studies	Requires rapid biomarker assessment; optimal timing unclear
Localized airway delivery	Increase lung exposure while minimizing systemic immunosuppression	Inhaled mAbs, nanoparticle carriers, siRNA targeting IL-1 pathway	Strong preclinical proof-of-concept	Delivery optimization; long-term pulmonary safety unknown
Integration with anti-alarmin biologics	Dual upstream blockade to control mixed granulocytic inflammation	TSLP, IL-33, IL-25 + IL-1 axis	Mechanistic rationale; no clinical validation for combination	Safety, cost, lack of trial data
Biomarker-enriched IL-1 targeting	Select patients with systemic/airway inflammatory burden for maximal therapeutic response	hs-CRP, IL-6, IL-1 signatures, exacerbation frequency	Supported by cardiovascular trial data (translational relevance)	Need for validated enrichment criteria in airway disease

COPD, Chronic Obstructive Pulmonary Disease; CRP, C-Reactive Protein; DPP-1, Dipeptidyl Peptidase 1; FeNO, Fractional Exhaled Nitric Oxide; G-CSF, Granulocyte Colony-Stimulating Factor; hs-CRP, High-Sensitivity C-Reactive Protein; IL-1, Interleukin-1; IL-1β, Interleukin-1 beta; IL-17 A, Interleukin-17 A; IL-25, Interleukin-25; IL-33, Interleukin-33; IL-6, Interleukin-6; ILC, Innate Lymphoid Cells; IRAK4, Interleukin-1 Receptor-Associated Kinase 4; mAbs, Monoclonal Antibodies; MyD88, Myeloid Differentiation Primary Response 88; NF-κB, Nuclear Factor kappa-B; NLRP3, Nod-Like Receptor Family Pyrin Domain Containing 3; siRNA, Small Interfering RNA; TNF-α, Tumor Necrosis Factor alpha; TSLP, Thymic Stromal Lymphopoietin

### Precision Medicine and Biomarker-Driven Stratification

One of the most promising strategies involves refined molecular stratification of patients. Future trials will likely benefit from selecting individuals with clear evidence of IL-1-driven inflammation, such as elevated sputum IL-1β, increased inflammasome gene signatures (e.g., upregulation of NLRP3 and caspase-1), neutrophilic airway inflammation, or relevant systemic inflammatory biomarkers [23, 37]. High-dimensional transcriptomic and proteomic profiling of sputum and airway samples can facilitate the identification of IL-1-high endotypes characterized by coordinated upregulation of inflammasome components, IL-1 family cytokines, and neutrophil-associated chemokines [37]. These molecular signatures are particularly relevant in severe, steroid-resistant asthma and neutrophilic COPD, where conventional therapies are often ineffective [22, 23].

Rather than targeting broad asthma or COPD populations, enrichment strategies focusing on patients with T2-low asthma, frequent exacerbations, or persistent neutrophilia despite maximal inhaled therapy may substantially improve the likelihood of demonstrating efficacy with IL-1 pathway inhibitors. Stratification using biomarkers such as blood eosinophils, fractional exhaled nitric oxide (FeNO), plasma IL-6, and sputum IL-1β can further refine appropriate patient selection [38]. Adaptive platform trial designs may accelerate identification of responsive subgroups by allowing simultaneous evaluation of multiple interventions within biomarker-defined populations and progressively refining molecular signatures [39].

### Upstream Inflammasome Inhibition

Given the central role of the NLRP3 inflammasome in IL-1β maturation, pharmacological inhibition of NLRP3 constitutes a compelling upstream strategy. By intervening upstream, this approach may overcome the compensatory cytokine redundancy that limited earlier IL-1-targeted therapies. Small-molecule inhibitors such as MCC950 have demonstrated robust suppression of neutrophilic inflammation in preclinical models of cigarette smoke exposure and steroid-resistant asthma, reducing IL-1β and IL-18 production, limiting pyroptosis, and improving airway hyperresponsiveness [40, 41]. However, MCC950 itself has been limited by developmental limitations due to hepatotoxicity and off-target effects [41]. Furthermore, some cryopyrin-associated periodic syndrome-related NLRP3 mutations confer resistance to MCC950, either through altered inhibitor binding or direct activation mediated by the pyrin domain of apoptosis-associated speck-like protein containing a caspase recruitment domain [42], highlighting the need for next-generation inhibitors with alternative binding mechanisms.

Next-generation NLRP3 inhibitors with improved safety and selectivity profiles (e.g., JT002, BAL-0028, OLT1177, LMT2368, ZAP-180013) are currently under clinical development for other inflammatory conditions and represent preclinical-to-early clinical translational candidates [43–45]. However, none have yet been validated in asthma or COPD.

### Dipeptidyl Peptidase 1 Inhibition

An alternative upstream strategy involves the inhibition of dipeptidyl peptidase 1 (DPP-1), also referred to as cathepsin C. Brensocatib, an oral, selective, reversible DPP-1 inhibitor, was approved by the FDA in August 2025 for the treatment of non-cystic fibrosis bronchiectasis [46]. DPP-1 plays a critical role in neutrophil biology by activating neutrophil serine proteases (NSPs), including neutrophil elastase, proteinase 3, and cathepsin G, during neutrophil maturation [47]. Notably, NSPs can directly process pro-IL-1β into its mature, biologically active form independently of the canonical inflammasome–caspase-1 pathway [48]. This supports a mechanistic rationale for DPP-1 inhibition to indirectly modulate IL-1β activity in settings of neutrophil-predominant inflammation, where multiple parallel activation routes contribute to IL-1β maturation.

The phase 3 ASPEN trial demonstrated a 21% reduction in the annualized exacerbation rate with brensocatib 10 mg over 52 weeks in patients with bronchiectasis, along with significant reductions in sputum NSP activity [49]. This represents clinically validated efficacy in bronchiectasis, a neutrophil-driven airway disease. However, the translation of these findings to asthma and COPD remains hypothetical and has not yet been evaluated in dedicated clinical trials. Given the mechanistic overlap in the mechanisms of neutrophil-driven inflammation among bronchiectasis, COPD, and neutrophilic asthma [50], DPP-1 inhibition may represent a promising adjunct or alternative strategy to direct IL-1β blockade or inflammasome targeting in selected patient subgroups characterized by neutrophil-predominant disease. Nevertheless, its role in asthma and COPD remains to be defined, and further clinical investigation is warranted to establish efficacy, optimal patient stratification, and long-term safety.

### Dual or Combinatorial Cytokine Targeting

The redundancy and complexity of inflammatory networks suggest that combination strategies may be required to achieve efficacy in patients with asthma or COPD. From an IL-1-centric perspective, dual or multi-cytokine targeting can be viewed as an extension of IL-1β modulation within a broader inflammatory network. Co-targeting IL-1β alongside other key cytokines involved in neutrophilic inflammation, such as IL-17 A, TNF-α, or IL-6, is supported by preclinical and translational evidence indicating that these cytokines act synergistically to sustain airway inflammation and neutrophil recruitment [51]. Since inhibition of individual cytokines often yields limited clinical benefit in patients with COPD, likely due to disease heterogeneity and the lack of biomarker-driven patient selection [52], these observations support the rationale for combination strategies. In COPD models, IL-17-neutralizing antibodies reduce neutrophilic inflammation and improve lung function, while IL-1β, IL-6, and G-CSF all contribute to neutrophil recruitment and tissue damage [53]. Combining IL-1 pathway inhibition with anti-IL-17 or anti-G-CSF approaches may therefore attenuate neutrophilic inflammation more effectively than single-target therapy.

However, despite this strong preclinical rationale, strategies such as bispecific antibodies, combination biologics, or multi-pathway inhibitors (e.g., JAK inhibitors) that illustrate the conceptual feasibility of broader cytokine blockade remain entirely experimental in asthma and COPD, with no demonstrated clinical efficacy to date. Careful patient phenotyping and biomarker-guided selection will be essential to balance potential efficacy with safety concerns, particularly the risk of infection associated with broader immunosuppression.

### Targeting IL-1 Receptor Accessory Pathways and Signaling Intermediates

Beyond ligand neutralization, interference with downstream signaling intermediates, such as myeloid differentiation primary response 88 (MyD88)-dependent pathways, interleukin-1 receptor-associated kinase 4 (IRAK4), or NF-κB activation, may offer alternative therapeutic targets. MyD88, an essential adaptor protein recruited to the activated IL-1R1 complex, and IRAK4, a serine-threonine kinase that propagates downstream signaling, are central to signal transduction from IL-1R1 and Toll-like receptors, culminating in NF-κB activation and amplification of pro-inflammatory cytokine production [54]. Targeting these shared signaling nodes could attenuate IL-1–driven inflammatory cascades while simultaneously modulating broader innate immune activation.

Small-molecule inhibitors targeting IRAK4, such as BI1543673, which is currently in development for systemic inflammatory diseases, attenuate IL-1-mediated amplification and neutrophilic inflammation in preclinical lung models and ex vivo human tissue, without completely abolishing innate immune signaling [55]. These agents remain in early clinical or preclinical development stages and have not yet been evaluated in asthma or COPD populations.

From a therapeutic translational perspective, modulation of these signaling intermediates may offer advantages over complete cytokine or receptor blockade by dampening excessive inflammatory amplification rather than fully abolishing upstream immune sensing. This partial inhibition strategy could be particularly relevant in COPD, where preserving antimicrobial host defense is critical given the high burden of infectious exacerbations. In this context, both MyD88 and IRAK4 inhibition must be carefully balanced, as complete disruption of these pathways, which are central to antibacterial immunity, may impair pathogen clearance and increase infection susceptibility [56]. Therefore, selective modulation rather than full blockade of IL-1R1 accessory signaling represents a more balanced paradigm, aiming to reduce chronic inflammatory amplification while maintaining essential mucosal immune competence [57].

### Modulating Innate Immune Cell Plasticity

Recent evidence linking IL-1β to ILC plasticity introduces another therapeutic concept focused on preventing maladaptive immune transdifferentiation. Interventions that stabilize ILC2 phenotype or inhibit the ILC2-to-ILC1/ILC3-like conversion may reduce maladaptive inflammation. Experimental studies suggest that IL-4 can reverse IL-12–induced ILC2-to-ILC1 conversion, while vitamin D3 abrogates IL-1β/IL-23/TGF-β–driven transdifferentiation toward IL-17-producing cells [18, 58]. These findings derive primarily from preclinical and in vitro experimental systems and have not yet been validated in clinical airway disease populations. Nevertheless, these findings indicate that targeting cytokine-driven plasticity (e.g., IL-12 or IL-18 blockade) or modulating metabolic and epigenetic regulators of ILC fate represents a potentially modifiable component of neutrophilic airway inflammation, particularly in steroid-resistant disease phenotypes [59, 60].

Similarly, interrupting IL-1β-induced epithelial and fibroblast transdifferentiation pathways may mitigate airway remodeling. IL-1β augments TGF-β1-induced epithelial-mesenchymal transition in bronchial epithelial cells, a process that is not abrogated by corticosteroids [9]. Epigenetic modulators or metabolic pathway inhibitors could potentially interfere with these structural reprogramming processes. Although still largely experimental, targeting IL-1β-driven immune and structural plasticity offers a mechanistically coherent approach to limiting neutrophil-predominated inflammation and disease progression.

### Temporal Targeting: Exacerbation-Focused Therapy

The temporal dynamics of IL-1β expression suggest that acute, short-term IL-1 inhibition during exacerbations may be more effective than chronic maintenance therapy. NLRP3 inflammasome activation and IL-1β levels are significantly elevated during acute exacerbations of COPD compared with stable disease and decrease as patients return to clinical stability [61]. In preclinical models of virus-induced exacerbations, IL-1β and IL-17 A are key mediators of neutrophilic inflammation, and IL-1 pathway blockade can significantly reduce neutrophil recruitment at the peak of viral replication [62].

These observations are primarily derived from preclinical and translational studies, and have not yet been confirmed in prospective clinical trials targeting exacerbation-specific IL-1β-driven endotypes in asthma or COPD.

Prospective trials specifically enrolling patients with an endotype associated with high levels of IL-1 and TNF-α during exacerbations may clarify whether temporally targeted intervention yields clinically meaningful benefits. Bafadhel and colleagues identified a pro-inflammatory exacerbation endotype defined by high airway concentrations of IL-1β and TNF-α, which is associated with bacterial infection and can be distinguished from viral, eosinophilic, and pauci-inflammatory endotypes [63]. Sputum IL-1α, IL-1β, and TNF-α have been validated as biomarkers for bacteria-associated exacerbations, supporting their use for patient selection in targeted therapeutic trials [64]. Furthermore, baseline airway IL-1β expression predicts future exacerbation risk and may mediate a vicious cycle between previous and future exacerbations, reinforcing the rationale for temporally targeted IL-1 inhibition in high-risk patients [4].

### Localized Airway Delivery

Systemic IL-1 blockade carries potential infection risks, particularly respiratory infections with *Streptococcus pneumoniae* and skin infections with *Staphylococcus* [65] and may not achieve optimal airway tissue concentrations [25]. Inhaled delivery of IL-1 pathway inhibitors or small-molecule inflammasome modulators could maximize local efficacy while minimizing systemic immunosuppression. Compared with systemic administration, inhaled mAbs offer faster onset, greater efficacy at lower doses, minimal systemic exposure, and lower risk of adverse events, although they may be more likely to induce the production of anti-drug antibodies, potentially limiting efficacy with repeated administration [66].

Although technically challenging, inhaled biologics and nanoparticle-based delivery systems are under development. Nanocarriers, including lipid nanoparticles, polymer nanoparticles, and liposomes, enhance pulmonary deposition, sustain drug release, and demonstrate potent anti-inflammatory effects in preclinical COPD and asthma models [67, 68]. For example, diacerein-loaded oleoliposome dry-powder inhalers targeting IL-1β/TNF-α suppress NF-κB-mediated inflammation in lung epithelial cells [68], while lipid nanoparticles delivering siRNA against pro-inflammatory cytokines achieve airway-specific gene silencing in asthma models [69].

Importantly, these inhaled nanoparticle-based strategies remain at the preclinical stage and have not yet been evaluated in clinical trials for asthma or COPD.

These advances suggest that localized airway delivery of IL-1 pathway inhibitors may represent a promising strategy to improve the therapeutic index by maximizing airway exposure while limiting systemic immunosuppression. However, clinical translation will require further optimization of nanoparticle design, chronic pulmonary safety assessment, and regulatory validation [67, 70].

### Integration with Emerging Biologics in Severe Asthma

Alarmins, including thymic stromal lymphopoietin (TSLP), IL-33, and IL-25, are epithelial cell-derived cytokines released from airway epithelial cells in response to environmental triggers such as allergens, microbes, and air pollution. Positioned upstream in the inflammatory cascade [71], they initiate and amplify immune responses by activating a broad spectrum of immune cells, including Th2 lymphocytes, ILC2s, and, to a lesser extent, Th1/Th17 cells [72]. Through these upstream effects, alarmins contribute to the development and maintenance of both eosinophilic (allergic) and neutrophilic (non-allergic) patterns of airway inflammation [73].

Anti-alarmin therapies may indirectly influence the IL-1 pathway through interconnected immunological mechanisms. First, IL-33 stimulation of dendritic cells promotes the production of IL-1β and IL-6, which are critical for Th17 cell differentiation and neutrophilic inflammation; therefore, blockade of IL-33 may attenuate this IL-1β-mediated pathway [74]. Second, dendritic cell-derived TSLP has been shown to negatively regulate IL-1β production during dectin-1 signaling, suggesting complex crosstalk between alarmin and IL-1 pathways [75]. Third, IL-1β enhances TSLP secretion from dendritic cells, indicating bidirectional regulation between these pathways [76]. Collectively, these mechanisms provide a biologically plausible framework for interaction between upstream alarmin pathways and IL-1 signaling. However, most of these mechanistic interactions are derived from preclinical or translational experimental systems, and their clinical relevance in human airway disease remains to be fully established.

From a theoretical perspective, dual upstream blockade, combining TSLP inhibition and targeting of the IL-1 pathway, may represent a strategy to address mixed granulocytic asthma by concurrently suppressing eosinophilic inflammation via alarmin inhibition and neutrophilic inflammation via IL-1 pathway blockade. However, despite this mechanistic rationale, no clinical trials or guideline-supported evidence currently exist for combined alarmin and IL-1 pathway targeting in asthma or COPD and its safety and efficacy remain unproven.

### Biomarker-Enriched IL-1 Targeting

Large-scale cardiovascular outcome trials of canakinumab, such as the CANTOS (Canakinumab Anti-inflammatory Thrombosis Outcomes Study) study, demonstrated that IL-1β inhibition reduces systemic inflammation and clinical events in patients with an elevated inflammatory burden, specifically those with a history of myocardial infarction and high-sensitivity C-reactive protein (hs-CRP) levels ≥ 2 mg/L [77]. The magnitude of clinical benefit observed in these trials was closely associated with both baseline inflammatory status and on-treatment reductions in hs-CRP and IL-6, underscoring the relevance of biomarker-guided patient selection in optimizing therapeutic efficacy [77].

It is important to distinguish between lung-centric and systemic inflammation when considering IL-1 pathway inhibition in airway diseases. Data from SARP demonstrate that systemic IL-6 inflammation, which is mechanistically linked to IL-1β, occurs in a subset of patients with asthma who have obesity and metabolic dysfunction, and operates independently of type 2 airway inflammation [78]. The PrecISE (Precision Interventions for Severe and/or Exacerbation-Prone Asthma) Network has tested whether IL-6 inhibition with tocilizumab improves asthma control in patients with systemic IL-6-related inflammation, recognizing that targeting the IL-1/IL-6 axis may benefit patients distinct from those with predominantly airway-centric inflammation [79].

Translating these findings to airway disease, biomarker-based enrichment strategies may improve the likelihood of response to IL-1 pathway inhibition in asthma and COPD. Candidate selection approaches may include elevated CRP, systemic inflammatory signatures linked to IL-1 activity, or frequent exacerbator phenotypes. Additionally, in asthma, biomarker-guided strategies incorporating blood eosinophils, FeNO, and CRP have already improved responses to biologic therapies [80]. Analogously, enrichment for IL-1–driven inflammation, whether predominantly systemic or airway-centric, may represent a rational strategy to optimize therapeutic responses to IL-1 inhibitors in selected airway disease populations.

## Conclusion

**Fig. 3:**
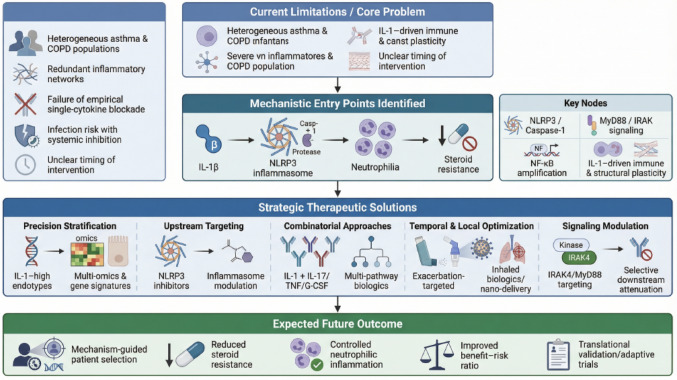
Schematic overview of the translational roadmap for IL-1β-targeted therapy in asthma and COPD. Current clinical limitations are linked to disease heterogeneity, limited efficacy of prior single-cytokine blockade, and inflammatory redundancy. Key mechanistic entry points include inflammasome activation, IL-1 receptor signaling intermediates, and downstream inflammatory amplification driving neutrophilia and steroid resistance. Strategic solutions encompass precision biomarker-driven stratification, upstream and combinatorial targeting, temporally optimized intervention, and localized delivery approaches. These integrated strategies aim to improve patient selection, enhance therapeutic efficacy, and reduce steroid resistance through mechanism-based intervention

IL-1β is a mechanistically central mediator in the inflammatory architecture of asthma and COPD, contributing to airway inflammation, neutrophil recruitment, and steroid resistance [2]. Yet the therapeutic potential of targeting this pathway remains unproven in patients, as the limited success of early IL-1-targeted trials underscores not the irrelevance of the pathway, but rather the challenges of applying single-cytokine blockade to heterogeneous and redundant immune networks without adequate molecular stratification [81]. To move the field forward, a shift from empirical targeting toward precision-defined intervention is required [82] (Fig. 3).

The therapeutic opportunities that have been outlined, including upstream inflammasome inhibition, biomarker-enriched patient selection, temporally targeted treatment during exacerbations, combinatorial cytokine modulation, modulation of signaling intermediates, and localized airway delivery, represent rational strategies to overcome prior limitations [83]. However, their clinical value remains to be validated through rigorously designed, mechanism-based trials [84]. Defining IL-1-high endotypes through integrated multi-omics profiling and inflammasome activation signatures will be essential to identify patients most likely to benefit from these approaches [82].

Future research should prioritize adaptive clinical trial designs enriched for biologically selected populations, alongside translational studies that link molecular signatures to treatment response and disease progression [82]. Equally important is the systematic evaluation of combination strategies and innovative delivery platforms to enhance efficacy while preserving host defense [83]. Airway gene expression of IL-1 pathway mediators, such as IL1R1, IRAK2, IRAK3, and Pellino E3 Ubiquitin Protein Ligase 1 (PELI1), has been shown to predict exacerbation risk in obstructive airway disease and may serve as biomarkers for patient selection in future trials [85].

Ultimately, the critical challenge is not to confirm that IL-1β contributes to airway disease, which is well established, but to determine how its pathogenic activity can be optimally therapeutically leveraged in a precise and context-dependent manner [2, 22]. This requires careful distinction between evidence derived from human studies and that originating from experimental models, as well as between clinically validated interventions and emerging preclinical hypotheses. If integrated with biomarker-driven stratification and innovative pharmacologic strategies, IL-1 targeting may evolve from an unsuccessful broad approach into a tailored intervention for well-defined inflammatory endotypes of asthma and COPD [80, 86].

## Data Availability

No datasets were generated or analysed during the current study.

## Abbreviations

COPD: Chronic obstructive pulmonary disease
CRP: C-reactive protein
CXCL: C-X-C motif chemokine ligand
DPP-1: Dipeptidyl peptidase 1
eDNA: Extracellular DNA
FeNO: Fractional exhaled nitric oxide
G-CSF: Granulocyte colony-stimulating factor
hs-CRP: High-sensitivity C-reactive protein
IFN-γ: Interferon-γ
IL: Interleukin
IL-1R1: Interleukin-1 receptor type 1
ILC: Innate lymphoid cell
IRAK: Interleukin-1 receptor–associated kinase
JAK: Janus kinase
LY6D: Lymphocyte antigen 6 family member D
mAb: Monoclonal antibody
MyD88: Myeloid differentiation primary response 88
NF-κB: Nuclear factor kappa-light-chain-enhancer of activated B cells
NLRP3: NOD-like receptor family pyrin domain containing 3
NSP: Neutrophil serine proteases
PELI1: Pellino E3 ubiquitin protein ligase 1
siRNA: Small interfering RNA
S100A8/A9: S100 calcium-binding protein A8/A9
T: Type (inflammation)
TGF-β: Transforming growth factor-beta
Th: T helper
TNF-α: Tumor necrosis factor-alpha
TSLP: Thymic stromal lymphopoietin
